# The clinical relevance of the presence of bridging syndesmophytes on kyphosis correction and maintenance following pedicle subtraction osteotomy for thoracolumbar kyphotic deformity in ankylosing spondylitis: a comparative cohort study

**DOI:** 10.1186/s12891-018-2013-y

**Published:** 2018-04-02

**Authors:** Sai-hu Mao, Zong-xian Feng, Bang-ping Qian, Yong Qiu

**Affiliations:** 1 Spine Surgery, the Affiliated Drum Tower Hospital of Nanjing University Medical School, Zhongshan Road 321, Nanjing, 210008 China; 2 Spine Surgery, Drum Tower Clinical Medical College of Nanjing Medical University, Nanjing, China; 3 Spine Surgery, Ningbo Medical Center Lihuili Eastern Hospital, Ningbo, China

**Keywords:** Bridging syndesmophytes, Pedicle subtraction osteotomy, Ankylosing spondylitis, Disc wedging, Correction loss

## Abstract

**Background:**

The presence of bridging syndesmophytes (BS) in spinal osteotomy region serves traditionally as one critical determinant for selection of osteotomy techniques. While nowadays the proportion of kyphotic ankylosing spondylitis (AS) patients receiving pedicle subtraction osteotomy (PSO) with yet mobile neighboring disc has seen a substantial increase. Literatures investigating the clinical relevance of the presence of BS on kyphosis correction and maintenance following PSO are scarce.

**Methods:**

A total of 71 thoracolumbar kyphotic AS patients treated with single-level PSO at our hospital between September 2010 and August 2014 were retrospectively reviewed, 32 of whom were stratified into the BS group (BG). The operative corrections of multiple spino-pelvic sagittal parameters were assessed. Comparison of the contribution of adjacent disc wedging to total correction per PSO segment was made between the BS and non-BS groups (NBG). The correction loss were also evaluated and compared with a minimum 2-year follow-up.

**Results:**

A significantly younger age (30.97 ± 8.28 vs. 40.31 ± 8.44 yrs., *p* < 0.001), smaller pelvic incidence (PI) (43.03 ± 10.60 vs. 49.36 ± 9.75°, *p* = 0.011), greater wedging index of osteotomized vertebra (1.17 ± 0.16 vs. 1.09 ± 0.08, p = 0.011) and larger local kyphosis (19.59 ± 10.84 vs. 13.56 ± 8.50°, *p* = 0.013) was observed in NBG preoperatively. Patients in BG and NBG accomplished comparable amount of kyphosis correction per PSO segment (40.22 ± 7.09 vs. 43.85 ± 8.71°, *p* = 0.062). However, the contribution of adjacent disc wedging to total correction per PSO was significantly larger in NBG [8.10 ± 6.19 (18.5%) vs. 1.09 ± 2.88° (2.7%), *p* < 0.001]. By ultimate follow-up, the global kyphosis (18.26 ± 10.97 vs. 21.51 ± 10.89°, *p* < 0.05) and thoracic kyphosis (37.95 ± 11.87 vs. 42.87 ± 11.56°, *p* < 0.05) deteriorated significantly in the NBG but not BG, so was further pelvic retroversion as represented by increased pelvic tilt (19.46 ± 8.13 vs. 23.44 ± 8.19°, *p* < 0.05) and decreased sacral slope (23.02 ± 9.12 vs. 18.62 ± 10.10°, *p* < 0.05). Loss of corrections concerning contribution of adjacent disc wedging was also larger in NBG (1.41 ± 3.27 vs. 0.22 ± 1.49°, *p* < 0.05).

**Conclusions:**

Our study might suggest that the evaluation and treatment methods of kyphotic AS patients needed to be fine-tuned with appropriate subgrouping by the presence of syndesmophytes with bamboo sign as they were potentially distinct groups with different PI, contributor of lordosing capability and prognosis that might require separate analysis.

## Background

Spinal lordosing osteotomy is a definitive treatment for symptomatic disabling thoracolumbar kyphosis secondary to ankylosing spondylitis (AS), and can achieve a high degree of relief from crippling symptoms [1–4]. The traditional cut-off rule for selection of osteotomy techniques is pedicle subtraction osteotomy (PSO) by presence of bridging syndesmophytes (BS) in osteotomy region; otherwise the Smith-Petersen osteotomy (SPO) is favored [1, 5, 6]. However, secular trends towards utilizing PSO rather than SPO for restoration of satisfactory and harmonious spinal-pelvic alignment may now be evident, and are predominantly linked to the powerful and sustained lordosing capability of PSO [1, 2, 7, 8].

PSO can be merely a closing wedge osteotomy (CWO) or strengthened to be a combined closing-opening wedge osteotomy (COWO) if a relatively larger amount of lordosing correction at one level is warranted, [1, 5] the extreme form of which has been called “the Y shape osteotomy” [9]. The key difference lies in that CWO is a shorting osteotomy utilizing the anterior cortex as the pivot, while for COWO, the middle column acts as the hinge, and opening and lengthening of the anterior column results in extra kyphosis correction [1, 9]. Sometimes these modified PSO procedures are beneficial for eliminating the necessity of performing additional osteotomy, and are thus favored to serve as a solution for the gray zone between single and two-level PSO.

With sufficient elasticity in the neighboring disc, the site of the anterior opening, if necessary, can resemble that of SPO, being in the intervertebral disc, and an extra degree of lordosing effect can be achieved through wedging of the adjacent discs during PSO procedures. However, when all the anterior longitudinal ligaments (ALL) are fully ossified, namely bamboo spine, elasticity is absent and the discogenic lordosing effect is hindered. In such cases, anterior opening may only occur when fracture of the anterior cortex happens or, less commonly, disruption of the bridging syndesmophytes develops. Thus the existence of syndesmophytes with bamboo sign in osteotomy region may significantly impact the lordosing capability per PSO segment, and subsequently lead to notable morphological transition of osteotomized vertebra (OV) between CWO and COWO. Despite being anticipated and frequently encountered, the exact impact of BS on lordosing capability in PSO remains to be elucidated quantitatively.

Earlier studies rarely mentioned such themes while a better understanding of the merits of neighboring disc wedging might be essentially helpful for osteotomy design and manipulation. This study sought to elucidate the clinical relevance of the presence of bridging syndesmophytes within osteotomy region on lordosing effect and prognosis of PSO by comparing the amount of kyphosis correction and maintenance between two different AS subgroups as stratified by the presence of BS neighboring the OV with a minimum 2-year follow-up.

## Methods

### Subjects

After ethics approval was obtained from the hospital review board, a comprehensive retrospective review of clinical records and radiographic data was performed on AS-related thoracolumbar kyphosis surgically treated in our hospital from September 2010 to August 2014. The indications for corrective osteotomy surgery were hindered forward gazing, poor cosmetic appearance, early fatigue and back pain, compression of the viscera and lastly restricted personal hygiene [10–12]. The necessary enrollment criteria were as follows: (1) thoracolumbar/lumbar kyphosis with apex locating at or below T11; (2) PSO performed at lumbar vertebrae; and (3) follow-up period exceeding 2 years. The following exclusion criteria were applied: (1) additional polysegmental SPO, continuous or skipped two-level PSO; (2) PSO through pseudarthrosis and (3) unilateral and asymmetrical syndesmophytes neighboring the OV. For those who fulfilled the inclusion and exclusion criteria, the patient demographic information were recorded including sex, age by surgery, apex location, level of osteotomy, length of instrumentation, ossification status of ALL neighboring OV, postoperative neurological status and surgical complications.

### Osteotomy planning and execution

PSO procedures were favored even for those with mobile anterior discs, and were principally performed in the lumbar vertebrae. The osteotomy level was usually 1–2 levels distal to the apex of kyphosis if the apical vertebra located in the thoracolumbar region (T11-L1). When the apex of kyphosis located at L2 or lower, the osteotomy vertebra being apex was the priority selection. These principles helped striking a balance between correction of regional kyphosis and restoration of lumbar lordosis during one-level PSO surgery. Moreover, a lower level of osteotomy should always be considered when more correction of SVA was warranted to restore the optimal sagittal balance. The length of instrumentation was usually 2–4 pairs of pedicle screw fixation in the cranial spinal segments and 2–3 pairs in the caudal parts. Adjustments would be made when there existed proximal or distal pseudarthrosis that required additional fixation.

Intraoperatively, once the pedicle screws were properly inserted at the planed vertebral segments and the wedging transpedicular osteotomy was adequately performed, the closure of the osteotomy gap was initiated by gradually straightening the special 4-poster spinal frame. Subsequently, the sagittal alignment was judged by whether the patient’s shoulder and pelvis were in the same horizontal line [1]. If the shoulder and pelvis were unparallel, an opening osteotomy would be preceded by pushing the osteotomy site manually and compressing the adjacent pedicle screws. The sound of a crack was indicative of the anterior column opening by fracturing the anterior cortex. This procedure could introduce an extra correction of approximately 10° [1].

### Radiographic evaluation

**Fig. 1:**
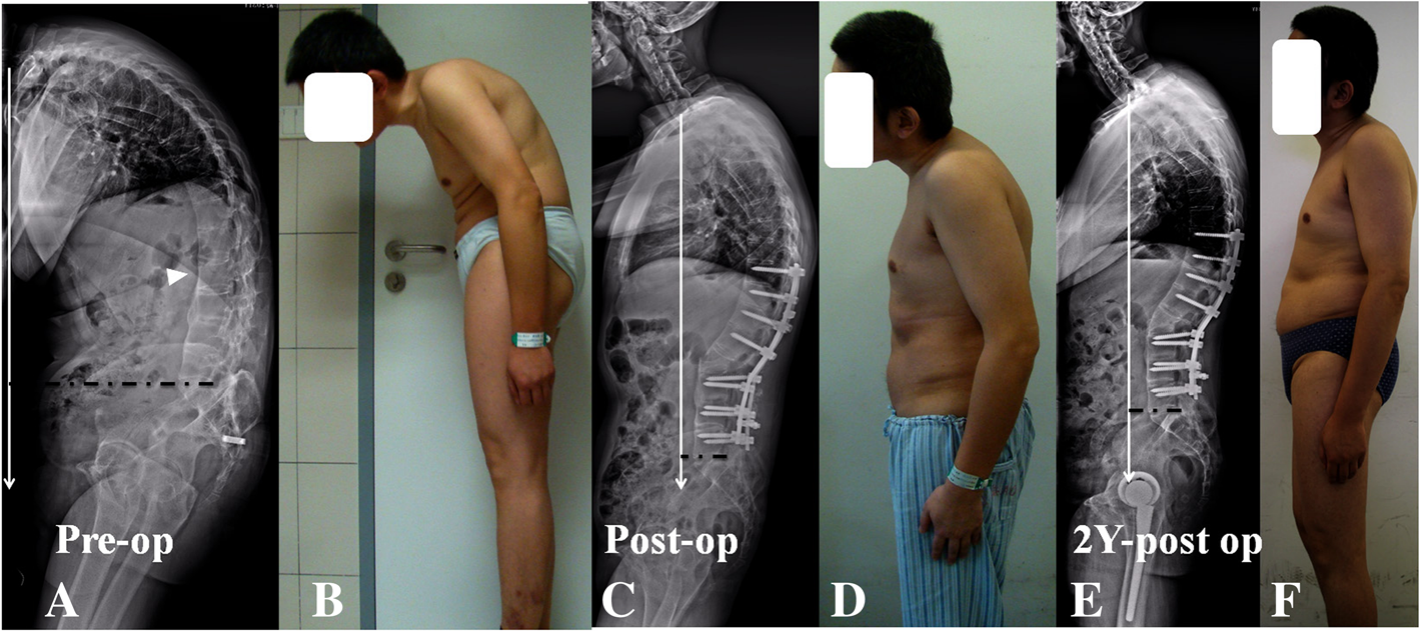
**a**, **b** 36-year-old AS patient, with circumferential ossification of ALL along multiple segments causing a long sweeping thoracolumbar kyphosis (GK: 91°, TK: 57°, LL: 11°, SVA: 22.9 cm, SS: 10°, PI: 42°). Following a PSO at L2 level, significant pre to post-operative improvements were detected concerning correction of GK, LL, SS and SVA (GK: 36°, TK: 54°, LL: -35°, SVA: 4.0 cm, SS: 16°). The contribution of adjacent disc wedging to total correction per PSO was 0% (**c**, **d**). At final follow-up, the obtained correction remained stable (GK: 40°, TK: 53°, LL: -34°, SVA: 6.5 cm, SS: 17°) (**e**, **f**)

**Fig. 2:**
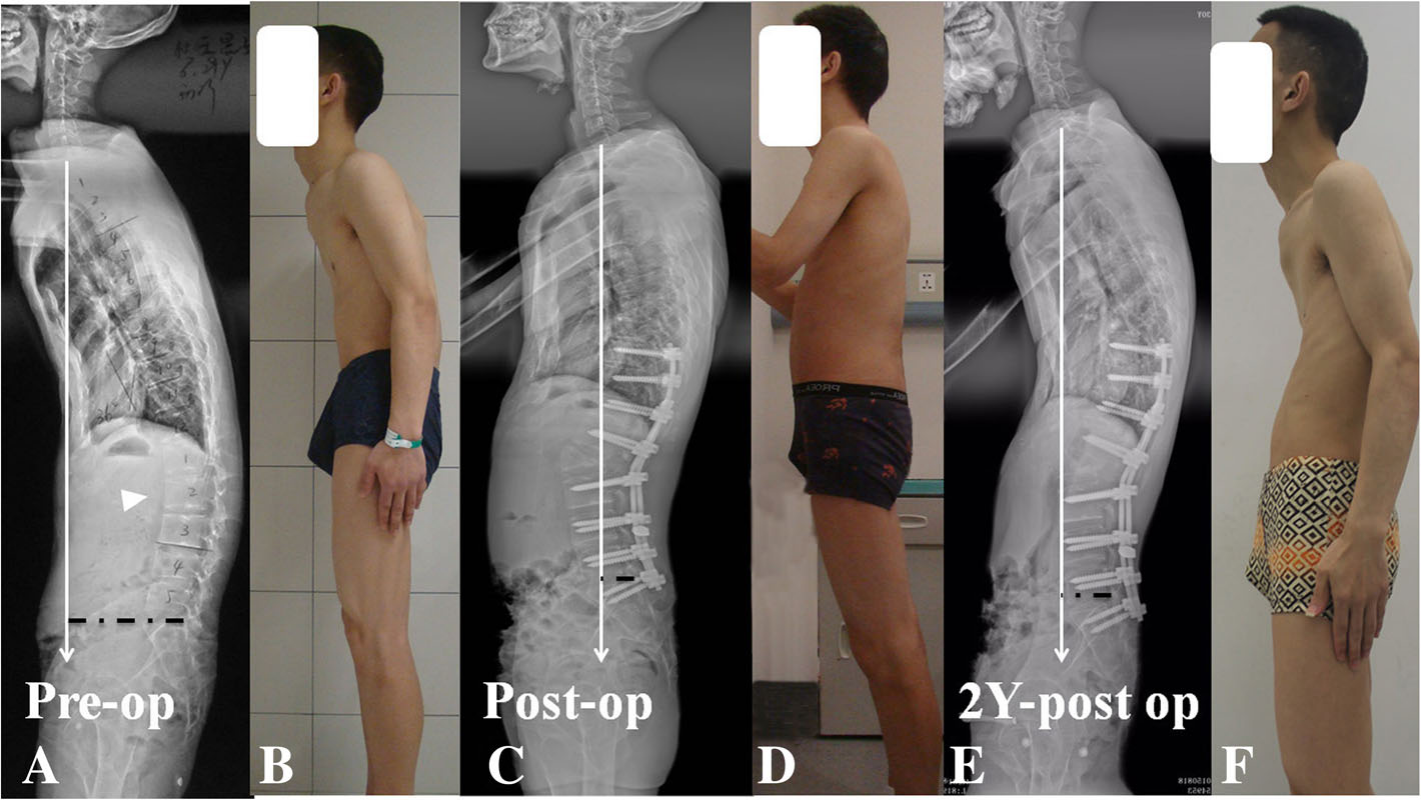
**a**, **b** 28-year-old AS patient, with rigid thoracolumbar kyphosis yet the ALL remained unossified (GK: 45°, TK: 12°, LL: 5°, SVA: 12.6 cm, SS: 4°, PI: 36°). Following a PSO at L2 level, significant pre to post-operative improvements were detected concerning correction of GK, LL, SS and SVA (GK: 15°, TK: 14°, LL: -34°, SVA: 0.8 cm, SS: 19°). The contribution of adjacent disc wedging to total correction per PSO was 28.3% (**c**, **d**). At final follow-up, the GK, TK, SS and SVA deteriorated (GK: 35°, TK: 33°, LL: -35°, SVA: 5.6 cm, SS: 11°) (**e**, **f**)

**Fig. 3:**
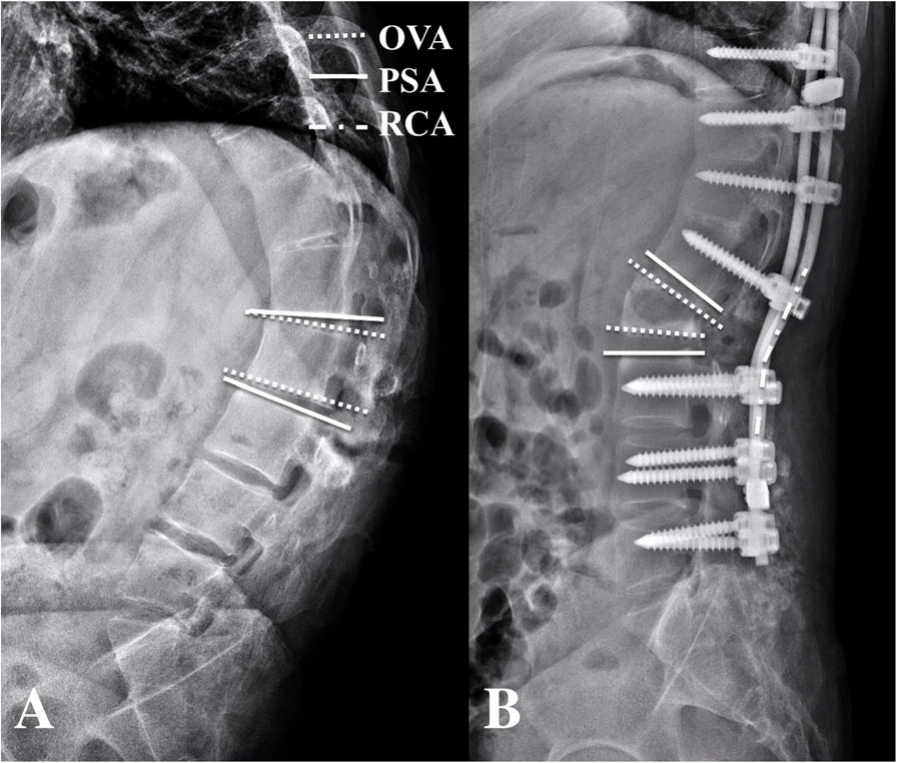
Illustration of the measurements of osteotomized vertebra angle (OVA) and pedicle subtraction angle (PSA) pre and post operatively (**a**, **b**) and rod contouring angle (RCA) (**b**)

The radiographic assessment was performed using the PACS (Picture Archiving and Communications Systems, PACS) workstation with standing lateral radiographs of the entire spine taken before surgery, before discharge and at the final follow-up. The patients were classified into either bridging syndesmophytes or non-BS group (BG, Fig. 1a or NBG, Fig. 2a) based on the pre-operative presence of ossified ALL neighboring the osteotomy vertebra. Multiple radiological spino-pelvic sagittal parameters were assessed preoperatively, postoperatively and at the final follow-up including global kyphosis (GK), thoracic kyphosis (TK), local kyphosis (LK) being centered at OV, lumbar lordosis (LL), sagittal vertical axis (SVA), spino-sacral angle (SSA), [13] T1 pelvic angle (TPA), [14] pelvic incidence (PI), pelvic tilt (PT) and sacral slope (SS) [1]. In order to analyze and compare the bony- and discogenic lordosing effect quantitatively, additional sagittal parameters regarding the wedging of vertebra and disc were proposed (Fig. 3a, b): (1) Osteotomized vertebra angle (OVA): the angle formed by the superior and inferior endplates of the osteotomized vertebra; [1] (2) The pedicle subtraction angle (PSA): the angle formed by the caudal endplate of 1 suprajacent vertebra above the osteotomy vertebra and the cranial endplate of 1 infrajacent vertebrae below the osteotomy vertebra; [15] and (3) Adjacent disc wedging angle (ADWA): defined as the sum of angulations formed between the upper and lower endplates of adjacent discs, which was equal to PSA minus OVA. The total lordosing effect per PSO segment was called as osteotomy angle and calculated as the difference between pre- and post-operative PSA (△PSA). The bony lordosing effect contributed by vertebral osteotomy was calculated as the post-operative OVA being subtracted by pre-operative OVA (△OVA), and the residual correction angle as compared to △PSA was considered to be attributed to disc wedging. The wedging index of OV was also evaluated and defined as the ratio of posterior to anterior height of vertebral body. Considering the potential role of rod contouring on anterior osteotomy opening, the disc opening in particular, the rod contouring angle (RCA) was also measured and defined as the supplementary angle to the angle formed between the proximal and distal rods converging at the osteotomy level (Fig. 3b). All the measured angles were regarded to be positive if it tilted backward, otherwise it was negative.

### Statistics analysis

Data were statistically analyzed using the SPSS software 17.0 (SPSS, Inc., USA). Descriptive statistics was performed to analyze patients’ demographics. Quantitative variables were presented with the mean and standard deviation (SD). Paired-sample t test was applied to compare the operative changes of multiple spino-pelvic sagittal parameters. Comparisons between BG and NBG were made using independent sample T test. The level of significance was set at *p* < 0.05.

## Results

A total of 71 patients fulfilling the above-mentioned inclusion and exclusion criteria were reviewed. There were 65 males (91.5%) and 6 females (8.5%). The average age was 35.2 ± 9.5 years (range from 17 to 59 yrs). Among these patients, 32 were stratified into the BG. The location of kyphotic apex was T11 for 7 cases; T12 for 22 cases; L1 for 25 cases; L2 for 14 cases and L3 for 3 cases. The osteotomy vertebrae being designated as apex+ 1 accounted for the largest share in total PSO (29 no., 40.8%), followed by apex (16 no., 22.5%), apex+ 2 (14 no., 19.7%), apex+ 3 (8 no., 11.3%) and apex-1 (4 no., 5.7%). Notably, majority of the PSO was performed at L2 level (44 no., 62.0%). The averaged number of fused levels was 8.3 (range, 5–12). The follow-up period averaged 34.3 ± 14.0 months (range, 24–96 months) for the total cohort.

### Radiological outcomes immediately after surgery

The radiological assessments of spino-pelvic sagittal parameters before and after surgery were summarized in Table 1. Significant pre to post-operative improvements were observed in terms of correction of GK, TK, LL, PT, SS, TPA, SSA and SVA (all *p* < 0.05). The total kyphosis correction obtained per PSO segment (△PSA) was 42.2 ± 8.2°. The averaged contribution of vertebral and adjacent disc wedging to total correction per PSO segment was 88.4% (37.3 ± 7.2°) and 11.6% (4.9 ± 6.1°), respectively.

**Table 1:** Radiological comparisons of spino-pelvic sagittal parameters before surgery, before discharge and at a minimum 2-year follow-up

Parameters	Groups	Preoperative	Postoperative	Final Follow-up
SVA(mm)	NBG	147.46 ± 58.92	34.46 ± 37.53*	34.17 ± 35.33
	BG	174.43 ± 51.59^†^	54.03 ± 44.99*	57.28 ± 35.18
SSA(°)	NBG	80.56 ± 16.68	108.36 ± 11.82*	106.18 ± 11.48
	BG	80.81 ± 14.20	104.19 ± 7.95*	103.84 ± 8.37
TPA(°)	NBG	44.41 ± 11.50	17.87 ± 8.54*	21.69 ± 8.71^#^
	BG	50.90 ± 12.13^†^	26.78 ± 10.11*	27.97 ± 10.30
GK(°)	NBG	66.28 ± 17.80	18.26 ± 10.97*	21.51 ± 10.89^#^
	BG	71.28 ± 16.14	22.09 ± 17.20*	21.63 ± 14.97
LK(°)	NBG	19.59 ± 10.84	−25.53 ± 8.76*	− 25.67 ± 10.38
	BG	13.56 ± 8.50^†^	−26.75 ± 7.35*	− 27.09 ± 7.36
TK(°)	NBG	41.87 ± 16.46	37.95 ± 11.87*	42.87 ± 11.56^#^
	BG	49.69 ± 16.78	46.63 ± 14.62*	44.41 ± 12.92
LL(°)	NBG	3.87 ± 14.39	−40.54 ± 14.06*	− 39.69 ± 12.45
	BG	0.22 ± 13.61	−38.44 ± 10.87*	− 38.94 ± 10.80
SS(°)	NBG	7.36 ± 9.21	23.02 ± 9.12*	18.62 ± 10.10^#^
	BG	8.75 ± 7.53	20.31 ± 6.26*	19.63 ± 6.69
PT(°)	NBG	35.64 ± 8.73	19.46 ± 8.13*	23.44 ± 8.19^#^
	BG	40.63 ± 10.28^†^	26.91 ± 9.42*	28.28 ± 9.59

^†^Indicates a statistically significant difference intergroup comparing preoperative values (*p* < 0.05)

*Indicates a statistically significant difference intragroup comparing preoperative and postoperative values (*p* < 0.05)

^#^Indicates a statistically significant difference intragroup comparing postoperative and final follow-up values (*p* < 0.05)

*SVA* indicates sagittal vertical axis, *TPA* T1 pelvic angle, *SSA* spinosacral angle, *GK* global kyphosis, *LK* local kyphosis, *TK* thoracic kyphosis, *LL* lumbar lordosis, *SS* sacral slope, *PT* pelvic tilt, *BG* bridging syndesmophytes group, *NBG* non-bridging syndesmophytes group

### Comparisons between BG and NBG

The BG and NBG were equivalent with regard to preoperative GK, TK, LL, SS and SSA (all *p* > 0.05) but not SVA, LK, TPA and PT (*p* < 0.05, Tables 1 and 2). A significantly younger age (30.97 ± 8.28 vs. 40.31 ± 8.44 yrs., *p* < 0.001) with smaller PI (43.03 ± 10.60 vs. 49.36 ± 9.75°, *p* = 0.011) was observed for patients in NBG. The wedging index of OV was also significantly larger in NBG (1.17 ± 0.16 vs. 1.09 ± 0.08, *p* = 0.011, Table 2), and was mirrored by a larger OVA in NBG (6.2 ± 4.6 vs. 3.8 ± 3.5°, *p* < 0.05). The disc wedging, being weighted by ADWA, was larger in NBG as well (3.0 ± 5.5 vs. 0.5 ± 4.7, *p* < 0.05). Patients in BG and NBG accomplished comparable amount of kyphosis correction per PSO segment (△PSA) (*p* > 0.05, Table 2). However, the contribution of adjacent disc wedging to total correction per PSO (△ADWA) was significantly larger in NBG [8.1 ± 6.2° (18.5%) vs. 1.1 ± 2.9° (2.7%), *p* < 0.001, Table 2]. This was in line with a significantly larger △OVA in BG (*p* < 0.05, Table 2). The averaged RCA tended to be larger in NBG but did not reach statistical significant difference (20.03 ± 7.27 vs. 18.36 ± 8.28°, *p* > 0.05). The length of instrumented segments was similar between two groups (*p* > 0.05, Table 2).

**Table 2:** Comparisons of patient demographic information, lordosing capability and correction loss per PSO segment between NBG and BG

Parameters	NBG	BG	t	p
Age	30.97 ± 8.28	40.31 ± 8.44	−4.687	0.000*
PI(°)	43.03 ± 10.60	49.36 ± 9.75	2.603	0.011*
LK(°)	19.59 ± 10.84	13.56 ± 8.50	−2.56	0.013*
Wedging index of OV	1.17 ± 0.16	1.09 ± 0.08	−2.635	0.011*
Instrumented segments	8.43 ± 1.25	8.06 ± 1.29	1.232	0.222
RCA(°)	20.03 ± 7.27	18.36 ± 8.28	−0.894	0.374
△PSA(°)	43.85 ± 8.71	40.22 ± 7.09	−1.895	0.062
△OVA(°)	35.74 ± 7.15	39.13 ± 6.99	2.003	0.049*
△ADWA(°)	8.10 ± 6.19	1.09 ± 2.88	−6.292	0.000*
Correction loss of △PSA(°)	2.62 ± 2.68	1.56 ± 2.11	−1.808	0.075
Correction loss of △OVA(°)	1.20 ± 2.14	1.34 ± 2.24	0.266	0.791
Correction loss of △ADWA(°)	1.41 ± 3.27	0.22 ± 1.49	−2.029	0.047*

*Indicates a statistically significant difference between the ossified and non-ossified groups (*P* < 0.05)

*OVA* indicates osteotomized vertebra angle, *ADWA* adjacent disc wedging angle, *PSA* pedicle subtraction angle, *RCA* rod contouring angle, *OV* osteotomized vertebra, *PI* pelvic index, *LK* local kyphosis

### Results of a minimum 2-year follow-up

By the ultimate follow-up, the global kyphosis (18.26 ± 10.97 vs. 21.51 ± 10.89°, *p* < 0.05) and thoracic kyphosis (37.95 ± 11.87 vs. 42.87 ± 11.56°, *p* < 0.05) deteriorated significantly in the NBG but not BG when compared with those data immediately after surgery (Table 1), so was the further pelvic retroversion as represented by increased pelvic tilt (19.46 ± 8.13 vs. 23.44 ± 8.19°, *p* < 0.05) and decreased sacral slope (23.02 ± 9.12 vs. 18.62 ± 10.10°, *p* < 0.05). Loss of corrections concerning △ADWA in NBG, as compared to that of BG, was also statistically significant larger (1.41 ± 3.27 vs. 0.22 ± 1.49°, *p* < 0.05). The correction loss of △PSA and △OVA were comparable between two groups (*p* > 0.05). The emerge of ossified ALL with resultant bridging syndesmophytes developed in 8 patients in NBG by the ultimate follow-up, and the incidence reached 20.5%. The representative surgical and follow-up changes for AS patients with and without BS were shown in Fig. 1 and Fig. 2, respectively.

### Complications

No vascular or severe neurological complications occurred in this patient cohort. Five patients experienced dural tear and cerebrospinal fluid leaks due to adhesions of dura to the ossified ligamentum flavum. They were treated by compression bandage and recovered uneventfully. Screw misplacement was confirmed in 5 cases with 7 medial perforations and 3 lateral perforations. Postoperatively, 2 patients developed transient brachial plexus injury, which recovered fully at 2 weeks and 3 weeks follow-up, respectively. Revision surgeries were performed for two patients with rod fracture from each group during follow-up, both of whom showed standard morphological appearances of OV. Two patients experienced traumatic cervical fracture with resultant neurological impairments and underwent emergency decompressive surgeries. Chin-on chest deformity was noted in one case in NBG at the follow-up of 72 months and necessitated cervical osteotomy to restore the horizontal gaze. Asymptomatic proximal junctional kyphosis was observed for one patient, who was advised to perform a meticulous follow-up.

## Discussion

Ossification of the paraspinal ligaments and joint capsules in AS are essential elements contributing significantly to ankylosis and kyphosis of the spinal column, following which a constellation of debilitating symptoms can appear and necessitate surgical intervention [16–20]. However, the level of ossification of the zygapophyseal joints and ligamentous apparatus can vary substantially by the time point of osteotomy surgery, from being restricted to the posterior spinal column to widespread syndesmophyte formation attacking all 3-column spinal structures, typically being a bamboo spine [21]. This discrepancy in the level of osteoproliferation can largely influence the determination of osteotomy strategy. Usually, an ankylosed kyphotic spine without bridging syndesmophytes is likely to be treated with SPOs, [5, 22] otherwise, various permutations in osteotomy techniques involving single level PSO procedure, single level PSO procedure combined with polysegmental SPOs, continuous or skipping 2-level PSOs will be considered [1, 23].

Of them, PSO is the mainstream type of effective osteotomy for AS [1, 2, 5, 7, 8, 13, 24]. Earlier studies have demonstrated a broad spectrum of lordosing effect ranging from 25°-36° for single-level PSO [1, 6]. This is usually sufficient for most AS patients, yet may be surpassed by when a cascade of compensatory mechanisms further strengthening the lordosing effect may be triggered. Anterior opening and wedging at the neighboring disc level usually serves as the first remedial action, followed by fracture and opening of the anterior vertebral column using modified PSO techniques, [1, 9] and finally the sagittal translational subluxation [25]. The role of the latter two types of mechanisms had been well described in the literature, [1, 9, 25, 26] while there was a dearth of research quantitatively analyzing the disc originated lordosing effect in PSO procedures. Theoretically, a PSO with two mobile neighboring discs could achieve correction approaching to that of a PSO with two additional SPOs. Thus a better understanding of this issue might be of paramount importance for the design of osteotomy strategy, particularly for those AS patients with sufficient elasticity in neighboring disc levels. Additionally, kyphotic AS patients with entirely different ossification status of ALL by time of spinopelvic sagittal imbalance requiring surgical intervention might be two distinct subgroups, and their prognosis following PSO might need to be elucidated separately, which was also the point of focus in this study.

The demographic information of this study initially revealed a significantly younger age with smaller PI for patients in NBG. It is well accepted that the capability to compensate for spinal kyphosis by retroversion of the pelvis was limited by the value of PI [27–29]. Thus this compensative mechanism could be surpassed at a younger age for AS patients with a low PI, predisposing to an early onset of sagittal imbalance by when the ALL being mostly not fully ossified. Additionally, the spinal segmental compensation was less limited in NBG, which was mirrored by the observed significantly larger LK along with more severe wedging of vertebrae in apical regions in NBG. As to the lordosing effect, the results firstly revealed that the contribution of adjacent disc wedging to total correction per PSO couldn’t be ignored. A relatively smaller amount of bony wedging osteotomy could achieve sufficient and even larger kyphosis correction when the adjacent discs were mobile and rod bending was appropriate. Despite the averaged amount of lordosing effect being a little less than the that of a single segment SPO, which generally resulted in 10° of correction [12], this discogenic lordosing effect might be essentially helpful to decrease the need of performing additional osteotomy and improve the realignment of sagittal profile.

We thus considered that there existed a gradient of sequential kyphosis correction during osteotomy closure and can be divided into three major steps. Once the special bow-type frame was gradually straightened, the closing of osteotomy gap was initiated until the two cancellous surfaces of the vertebral osteotomy got touched tightly. If the patient’s shoulders didn’t reach the same horizontal line as the pelvis, adjacent disc wedging and opening could occur spontaneously or manually to further strength the lordosing effect unless the intervertebral discs were too stiff to be opened up anteriorly. If it was still insufficient to achieve the best sagittal alignment, the anterior cortex would be fractured and opened by manually pushing the osteotomy site until a sound of a crack could be heard. In most circumstances, these two modified opening PSOs would preclude the possibility of performing additional osteotomies.

This study also demonstrated that patients in NBG were more likely to lose correction with time as to TPA, GK and TK, while the SVA and LL were relatively well maintained. This was in line with the dynamic changes of the pelvis during follow-up, being expressed as the decreases in SS through pelvic retroversion, which were representative of the intervening compensative mechanisms of pelvis to compensate for the correction loss in the proximal spinal segments and prevent an increasingly SVA with time. The loss of disc wedging was also higher in NBG, yet the correction obtained through vertebral wedging remained relatively stable. In other words, the correction would be better maintained if osteotomy was performed on bamboo spine. This higher possibility of correction loss for patients in NBG may be due to the fact that patients in this subgroup mostly had earlier forms of AS with underlying active inflammation. And their osteoproliferation process progressed during follow-up, developing collapse of proximal and distal discs, decreasing the mobility of non-fused segments, increasing the kyphotic angle of non-instrumented segments involved in the global kyphosis, and finally detracting from the initial correction [24, 30]. This was also mirrored by the fact that 20.5% of the patients in NBG developed bridging syndesmophytes at the neighboring disc level by the ultimate follow up. As to the relatively more loss of correction in the disc levels of fused segments in NBG, it should be attributed to the strong biomechanical resistance against the maintenance of discogenic lordosing effect, arising from absence of bridging syndesmophytes. This could reduce the grip of screws gradually in osteoporotic spine, resulting in the loss of correction in the disc level of the instrumented area [24].

We should note that the AS-related kyphotic deformity was regarded to be best treated when the inflammatory activity were less pronounced or even absent [31]. However, the sagittal imbalance and the associated impairment of physical function that warranted surgical intervention might emerge far sooner, being in the intermediary stage, by when the anterior longitudinal ligaments and discs were usually not ossified, especially for patients with low PI. Thus the increasing number of AS patients requiring corrective PSO with underlying mobile discs could not be ignored and results of this study were beneficial for design of osteotomy strategy and clarify the prognosis for this particular patient cohort.

Limitation of this study lied in that the medical therapies during follow-up vary greatly among individuals. We were not sure if strict control of inflammation activity could fully prevent the correction loss. Further investigation should be carried out to clarify this issue.

## Conclusions

Judging from the overall aspects, we concluded that the evaluation and treatment methods of kyphotic AS patients needed to be fine-tuned with appropriate subgrouping by the presence of syndesmophytes with bamboo sign as they were potentially distinct groups with different PI, contributor of lordosing capability and prognosis that might require separate analysis. For subgroup with smaller PI, the timing of surgical intervention might be more likely at a younger age, by when the ALL was mostly not fully ossified and bridged. Correspondingly, the lordosing effect through adjacent disc wedging could occur and serve as an important remedy when that of the vertebral wedging was insufficient, and could be strengthened with appropriate closing osteoclasis and sufficient rod contouring. Otherwise, closing-opening wedge osteotomy should be primarily considered to magnify the lordosing effect, and if necessary, a second osteotomy should be performed. Finally, considering the relatively higher risk of correction loss during follow-up for patients with the ALL not being fully ossified, a strict and meticulous follow-up, particularly the monitoring of the ESR (erythrocyte sedimentation rate) and CRP (C-reactive protein) level, should be considered to guard the recurrence of underlying inflammation causing kyphosis progression.

## Abbreviations

ADWA: Adjacent disc wedging angle
AS: Ankylosing spondylitis
BG: Bridging syndesmophytes group
BS: Bridging syndesmophytes
GK: Global kyphosis
LK: Local kyphosis
LK: Local kyphosis
LL: Lumbar lordosis
NBG: Non-bridging syndesmophytes group
OV: Osteotomized vertebra
OVA: Osteotomized vertebra angle
PI: Pelvic incidence
PSA: Pedicle subtraction angle
PSO: Pedicle subtraction osteotomy
PT: Pelvic tilt
RCA: Rod contouring angle
SS: Sacral slope
SSA: Spinosacral angle
SVA: Sagittal vertical axis
TK: Thoracic kyphosis
TPA: T1 pelvic angle
